# Risk Factors for Post-cardiac Surgery Infections

**DOI:** 10.7759/cureus.31198

**Published:** 2022-11-07

**Authors:** Bandar A Alghamdi, Riyadh A Alharthi, Bayan A AlShaikh, Mohammed A Alosaimi, Abdulaziz Y Alghamdi, Nik Yusnoraini, Ali Almashhor

**Affiliations:** 1 Cardiac Surgery, King Fahad Armed Forces Hospital, Jeddah, SAU; 2 College of Medicine, Taif University, Taif, SAU; 3 College of Medicine, Ibn Sina National College for Medical Studies, Jeddah, SAU; 4 Institute for Research in Molecular Medicine, Universiti Sains Malaysia - Kampus Kesihatan, Kubang Kerian, MYS

**Keywords:** surgical site infections, sternal wound infections., deep sternal wound infection, postoperative wound infection, surgical wound infection

## Abstract

Background

Cardiac surgery infection is a life-threatening complication associated with high morbidity and mortality. One of the main types of these infections, surgical site infections (SSIs), also called postoperative wound infections basically delayed the post-surgical recovery in many patients. These infections rarely happen within 30 days after surgery due to different risk factors.

Objectives

The purpose of this study is to determine the risk factors that are involved in causing post-cardiac surgical infections.

Methods

This study was a retrospective study. The data of postoperative cardiac patients was obtained from the Cardiology and Cardiac Surgery Center in King Fahad Armed Force Hospital, Jeddah. Data on the patients were collected from 2016 to 2021. Eligible patients were those of age 18 and greater. These patients were evaluated on basis of the pre and postoperative risk factors and were analyzed for 30 days after discharge and those that developed SSIs were identified.

Results

Out of the total number of 2366 patients, 151 patients (6.4%) were identified with post-surgery infections out of which 107 (4.5%) had developed superficial wound infections (SSWIs) and 44 (1.9%) had developed deep wound infections (DSWIs). Infection mainly occurs in the male population (n=88, 58.3%). Major risk factors that were the main cause of post-cardiac surgery infections were diabetes (61.5%), hypertension (56.9%), smoking (38.4%), renal failure (27.1%), and re-do operation (25.1%).

Conclusion

Our study has demonstrated major risk factors that are involved in the occurrence of post-cardiac surgery infections like smoking, diabetes mellitus, sex, more than one operation during a single stay, etc. In the future, the contribution of various other factors involved in the occurrence of surgical site infections and best practices and methods should be studied and implemented to prevent the occurrence of post-cardiac surgery infections. Various simple techniques can still be utilized to prevent these sorts of infections, which will decrease the mortality rate.

## Introduction

In about 5-21% of cardiac surgery cases, infectious complications occur. These infections can lead to prolonged recovery after the operation and have resulted in an increase in the postoperative death rate by five times. An analysis had shown that 47% of the patients who had developed postoperative infections had delayed their stay in the hospital by 14 days as compared to non-infected patients [1]. This has caused an increase in the cost of care for these patients [2]. Despite that, there are several ways through which these postoperative infections can be reduced. It starts with extensive vigilance both during preoperative screening and postoperative care in the ICU. The most common areas where an operated patient can develop infections are devices or catheters (20.5-25.2%), surgical site (27.7%), and respiratory tract (45.7-57.8%) [3]. Surgical site infections (SSIs), also called postoperative wound infections, basically delayed post-surgical recovery in many patients. These infections normally happen at the part or site of the body where the operation was done within 30 days after surgery. These SSIs are primarily caused due to presence of microbes or pathogens on the patient’s skin or bacterial colonization within the genital tract or alimentary canal [4]. With the emergence of anti-microbial-resistant pathogens, it has now become a great challenge to treat SSIs [5].

SSIs can differ on the basis of their wide severity range and are of two types i.e. superficial wound infections (SSWIs) and deep wound infections (DSWIs). SSWIs involve pectoralis fascia, subcutaneous tissue, and skin, and around 0.5-8% of cases of post-cardiac surgery are complicated by this type of infection [6]. On the other hand, the incidence rate of DSWIs is less as compared to SSWIs, i.e. 0.4-2%, and usually involves the mediastinum, substernal space, and sternal bone [7]. However, these infections are considered to be the most dangerous type of SSIs because if they occur, they can increase the mortality rate [8] with a 50-80% higher actual incidence rate when undertaking post-discharge surveillance [9]. Post-surgical infections are the major cause of mortality and morbidity along with problems in the initial identification of SSIs [10]. Although the occurrence of SSIs had decreased by up to 2.7%, the mortality rate is still up to 25%. It was found that 4.3% of 2954 postsurgical patients developed SSIs [11]. The occurrence of SSIs after surgery poses a significant burden on resources and clinical utilization in the USA. It was disclosed that 1.21% (78,669) of patients developed SSIs after surgery [12]. These SSIs were found to be associated with various risk factors like the patient’s age, patient’s gender, patient’s race, pathogen attack, obesity, diabetes mellitus, previous disease (cardiac disorder, etc.), patient frailty, long-duration surgeries, and surgical complexities [13]. It was reported that 9.9% of post-surgical patients developed SSIs [14]. To control SSIs, various preoperative antibiotic prophylaxis, careful wound handling and treatment, and modern surgical techniques are now used. However, despite using these techniques, SSIs are still a major concern for post-cardiac surgery patients with a reported occurrence of 0.25-2.9% in one study [15]. The results of SSIs for post-cardiac patients can be devastating, including high costs, long duration of stay, and even death (10-29%) [16].

A study reported that 38 out of 1,268 patients (total sample population) were diagnosed with SSIs (3% occurrence rate). Out of these 38 SSIs, 18 were mediastinitis (1.4%) and 20 were superficial incisional infections (1.6%). A positive culture for pathogenic infections was found in 28 patients, and the most commonly isolated pathogen was found to be Staphylococcus. The National Nosocomial Infections Surveillance System (NNIS) risk index in post-cardiac operated patients was found to be 2 or greater than 2 (p<.004, relative risk=2.4). Various risk factors independent of SSIs were found in the patients, i.e. pericardial effusion or reoperation for cardiac tamponade and coronary artery bypass graft with the use of the internal mammary artery [17]. Similarly, another study was conducted on children to determine postsurgical infections after cardiac surgery. It was found that 713 children were scheduled for cardiac surgery, out of which 617 were asymptomatic and 96 were suffering from upper respiratory infections (URIs). The survey results had shown that children suffering from URIs had significantly higher post-surgical pathogenic infections (5.2%), respiratory problems (29.2%), and multiple complications (25%) as compared to asymptomatic children (1%, 17.3%, and 10.3%, respectively). These children had a delayed stay in the ICU (75.9 +/- 89.8 h) than asymptomatic ones (57.7 +/- 63.8h). However, their overall stay in the hospital was not that much different (7-8 days) [18]. Similarly, a study showed that of 2230 patients that had undergone cardiac surgery, 4.8% (n=107) were found to have SSIs while 4.7% (n=104) were suspected to have developed SSIs. It was found that these SSIs were associated with a delayed stay in the ICU (134.1h with 99168.2 CI in suspected patients while 266.1h with 231.6-300.7 CI in infected patients) and 30 days increased mortality ratio (3.7% for suspected patients and 6.6% for infected patients) [19]. A study concluded that the occurrence of SSIs was predominant in males as compared to females, i.e. 130 out of 144 patients were males and were suffering from SSIs (90.3%). Data analysis disclosed that factors that were involved in the development of post-coronary artery bypass graft (CABG) SSIs were lower serum cholesterol level (2.7%), higher blood urea N level (19.1%), and higher body mass index (BMI) (18.6%) [20]. Similarly, a study was conducted on 372 patients that had undergone cardiac surgery to evaluate what could be the contribution of patient care variables and different host factors that could aid in developing post-cardiac infections. Different host factors that were studied in this study included sex, age, type of operation, country of origin, functional cardiac status, and coronary artery bypass operations. It was found that a high risk for infection was associated with more than one operation carried out during a single stay at the hospital followed by a long-duration operation time for coronary artery bypass surgery (> 6 hours), and lastly, the age of the patients (65 years or of higher age). For patients that had undergone coronary artery bypass surgery, the incidence of chest wound infections was associated with host factors like their age and heart functions, whereas the duration of the operation had mostly resulted in an incidence of infections at the donor [21].

Similarly, another study was conducted that determined the risk factors that were involved in the occurrence of SSIs in post-cardiac operative patients, and the best measures were developed to prevent the occurrence of these SSIs. This study revealed that the risk factors that could normally be the cause of the incidence of SSIs were obesity, age, operation time, diabetes, surgical re-exploration, blood glucose control, emergency context, and preoperative showing differences from what is described in best-practice guidelines and blood transfusions. These factors played a major role in the development of infections. Sarah also proposed best practices that could be utilized in controlling these infections and that includes prior identification of patients that are at higher risk of developing infections, improvement in clinical documentation, targeting of areas to ensure care efforts, and improvement in adherence to practice manuals [22]. Another study was conducted to determine the types of infection and their outcomes in patients that had undergone cardiac surgeries. The study was conducted on 973 patients that had undergone cardiac surgeries. Twenty point three percent (20.3%; n=198) of the patients had developed post-surgery infections. Results had shown that out of these 198 patients, 1.7% suffered from DSWIs, 2.8% from bloodstream infections, 5.7% from SSWIs, and 9.1% from pneumonia. Major risk factors that were responsible for the occurrence of these infections included: long surgery duration, insulin-dependent diabetes mellitus (IDDM), age, the use of a surface-damaged transesophageal echocardiography (TEE) probe during operation, EuroScore II, and a reoperation for bleeding. Infections were also caused by pathogenic microbes, i.e. two patients were infected by Enterococcus faecalis leading to endocarditis, 10 patients were infected by Pseudomonas aeruginosa, and 22 patients by Klebsiella oxytoca (a multidrug-resistant strain). These three pathogenic strains were obtained from TEE probes, even after decontamination of probes, that were used during operations. A three-point two percent (3.2%) 30-day death rate was found in this patient cohort [23]. Similarly, a study was done to determine the frequency of occurrence of infections and the death rate in patients within 65 days after cardiac surgery. Only 5% of 5,158 patients suffered from post-cardiac surgery infections. Risk factors include heart failure (HR 1.47), prolonged surgery (HR 1.31), and chronic lung disease (HR 1.66). Prophylaxis with second-generation cephalosporins (HR 0.7) was associated with a decreased risk of infection. But on the other hand, the infection risk increased due to >48 hours of ventilation, >48 hours of postoperative antibiotic duration, 24-48 hours of incubation time, and stress hyperglycemia [24].

## Materials and methods

Study design

This study was a retrospective study. The data of postoperative cardiac patients was obtained from the Cardiology and Cardiac Surgery Center in King Fahad Armed Force Hospital, Jeddah. Data from the patients were collected from 2016 to 2021. Eligible patients were those of age 18 and greater. These patients were evaluated on basis of the pre and postoperative risk factors and were continuously analyzed for 30 days after discharge and those that developed SSIs were identified.

Study population and sampling methodology

In this study, the general population is Saudi patients who underwent cardiac surgery, either CABG, minimally invasive cardiac surgery (MICS), or valvular heart surgery, were included, and those who underwent noncardiac thoracic surgery, tracheoinnominate artery fistula ligation, tumor excision, pacemaker implantation, and aortopexy were excluded from the study. Also, the patients that died within 24-48 hrs of surgery were excluded, Both primary and secondary data sources were utilized. The primary data source was obtained via questionnaires and secondary through the medical records and history of the patients. The data were collected using questionaries in four categories: demographic data (gender, age, BMI) pre-surgical phase, medical variables (prior cardiac operation, smoking, etc.) during the pre-surgical phase, surgical data (type, number, time, length of stay, etc.) on the day of the operation and data results/outcomes (presence or absence of SSIs, etc.).

Potential risk factors that were analyzed were gender, age, DM, BMI, hypertension (HTN), smoking, liver disease, auto-immune disease (SLE, antiphospholipid), renal failure, cardiopulmonary bypass (CPB) time (Min), type of surgery, emergency, bilateral mammary artery, redo operation, re-opening, complex surgery, operative ICU stay (days), and on steroid.

Data analysis

Data analysis was done by using a Microsoft Excel spreadsheet (Microsoft Corporation, Redmond, WA) and SPSS software (IBM Corp., Armonk, NY). The descriptive type of statistical analysis includes means, standard deviation, and percentages while the inferential type of statistical analysis (univariate analysis) includes the student's t-test and chi-square test to assess different variables. Significance was considered at a p-value <0.05.

Ethical considerations

Ethical approval was provided by the Institutional Review Board (IRB) of King Fahad Armed Forces Hospital, Jeddah (REC-469).

## Results

**Figure 1:**
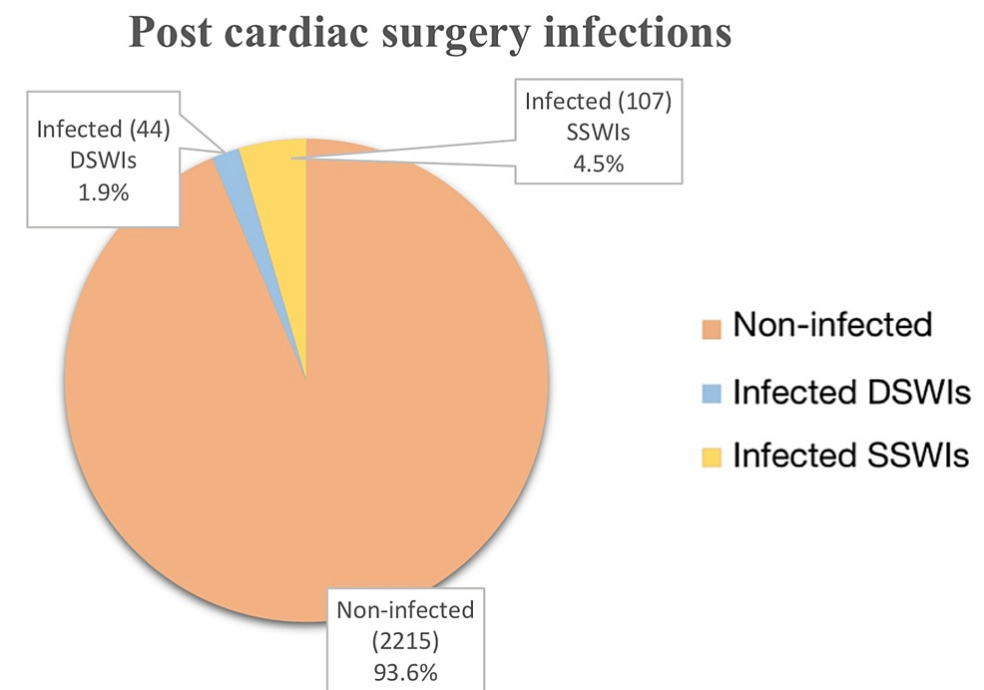
Number of patients that develop infections after undergoing cardiac surgery DSWI: deep wound infection; SSWI: superficial wound infection

Table 1 and Figure 1 showed that out of a total of 2366 patients who had undergone cardiac surgery, 151 were identified with SSIs, out of which 107 (4.5%) had developed SSWIs and 44 (2.2%) had developed DSWIs. 

**Table 1:** Number of patients that develop infections after undergoing cardiac surgery out of the total (n=2366) DSWI: deep wound infection; SSWI: superficial wound infection

Non-infected	Infected (151)	
	DSWIs	SSWIs
2215	44	107
93.6%	1.9%	4.5%

**Figure 2:**
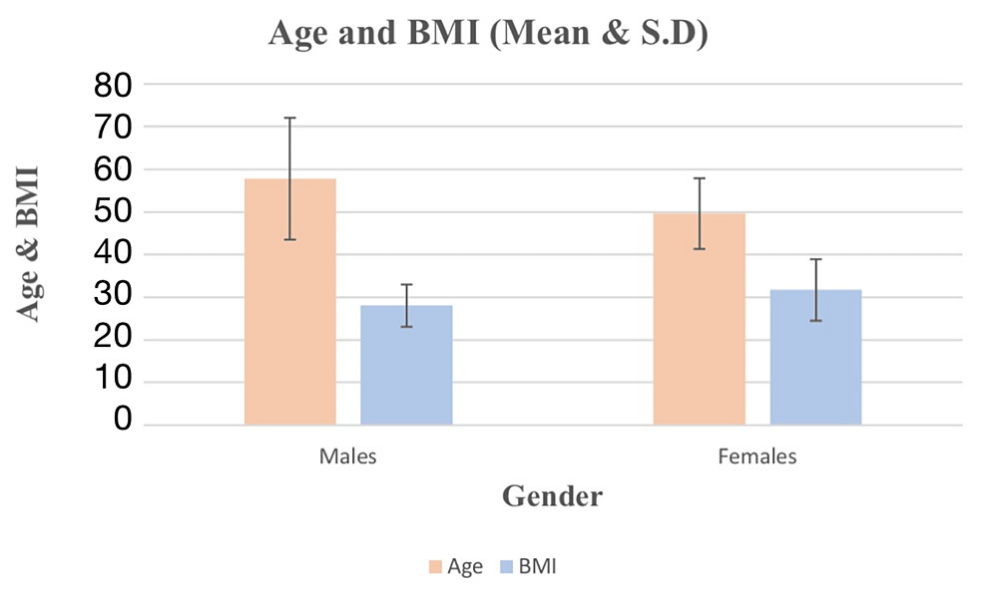
Mean age and BMI of the patients

Table 2 showed that the analysis revealed that out of 151 patients, 88 were males (58.3%) with a mean age and BMI of 58 ± 14 years and 28.01 kg/m^2^, respectively, and 63 were females (41.7%) with a mean age and BMI of 50 ± 8 years and 31.72 kg/m^2^, respectively, according to Figure 2. Thus, it is found that the prevalence of cardiac surgeries and post-cardiac infections was mainly in males as compared to females.

**Table 2:** Age and BMI of the patients

Gender	Males	Females
Number	88 (58.3%)	63 (41.7%)
Age		
Mean	57.81	49.61
S.D	14.26	8.29
BMI		
Mean	28.01	31.72
S.D	4.93	7.22

Table 3 shows that most of the participants (53; 35.1%) were within the age group of 50-60 years, and 42 (27.8%) were within the age group of 41-50 years. Thirty-one (31; 20.4%) were below 41 years and 25 (16.5%) were above 60 years old. About 88 (58.3%) were males, and 63 (41.7%) were females. In regard to the co-morbidities, 93 (61.5%) had DM, 86 (56.9%) were with HTN, and 42 (27.8%) of the participants had renal failure. According to smoking status, most of the patients smoked (81; 53.7%) and 60 (39.7%), whereas 10 (6.6%) used to smoke.

**Table 3:** Patient’s preoperative and clinical characteristics

Variables	No. of patients n(%)	Mean/SD
Gender		
Male	88 (58.3%)	
Female	63 (41.7%)	
Age groups		
<18 years	2 (1.3%)	53 (14.5)
18-30 years	10 (6.6%)	
31-40 years	19 (12.5%)	
41-50 years	42 (27.8%)	
50-60 years	53 (35.1%)	
61-80 years	20 (13.2%)	
>80 years	5 (3.3%)	
Co-morbidities		
DM	93 (61.5%)	
Renal failure	42 (27.8%)	
Autoimmune diseases	Not any	
HTN	86 (56.9%)	
Liver disease	Not any	
Smoking		
Smoking	81 (53.7%)	
No smoking	60 (39.7%)	
Used to smoke	10 (6.6%)	
Surgery type		
CABG	67 (44.3%)	
Others	84 (55.6%)	
Body mass index (BMI)		30 (6.58)
Emergency	Not any	
Complex surgery	Not any	
Operative ICU stay (days)		16 (8.7)
CPB	38 (25.1%)	
CPB time (min)		62 (37.97)

It was found that patients with DSWIs were not suffering from any sort of liver and auto-immune disease. There was no sort of emergency, no bilateral mammary artery, and there was no patient on steroids. One out of 44 patients had a complex surgery, as her sternal and femoral wounds were oozing. Out of 44 patients, 27 suffered from DM (61.36), 25 suffered from HTN (56.8%), 17 smoked (38.63%), 12 suffered from renal failure (27.27%), and 11 had more than 1 operation (25%). The patient’s characteristics are given in Table 4.

**Table 4:** DSWI patient’s characteristics; a univariate analysis RR: risk ratio (relative risk); DSWI: deep wound infection

	DSWI Patients (n=44)		Unadjusted RR	P-Value
	Males (n=26)	Females (n=18)		
Age (mean, SD)	57.8 (14.2)	49.6 (8.2)		<0.001
BMI (mean, SD)	28.01 (4.9)	31.7 (7.2)		<0.001
DM	20 (76.9%)	7 (38.8%)	1.98	0.011
HTN	18 (69.2%)	7 (38.8%)	1.78	0.045
Smoking	14 (53.8%)	3 (16.6%)	3.23	0.012
Renal failure	11 (42.3%)	1 (5.5%)	2.03	0.007
CPB time (min/SD)	53.9 min (34)	72.8 min (38)	0.64	0.04
Re-do operation	10 (38.45%)	1 (5.5%)	6.92	0.013
Operative ICU stay (days)	16.5 days (8.9)	15.4 days (7.5)		<0.001
Surgery type				0.54
CABG	13 (50%)	7 (38.8%)	1.23	
MVR+TVP	9 (34.6%)	8 (44.4%)	1.06	
MVP+TVP	3 (11.5%)	3 (16.6%)	0.50	
AVR	2 (7.6%)	0	0.1	

The same was the case with SSWI patients. No patient was on steroids or had liver or auto-immune disease. No one had had any complex surgery. Out of 107 patients, 66 suffered from diabetes mellitus (61.7%), 61 from hypertension (57%), 41 from smoking (38.3%), 29 from renal failure (27.1%), and 27 had more than one surgery (25.2%). Their characteristics are given in Table 5.

**Table 5:** SSWI patient’s characteristics; a univariate analysis RR: risk ratio (relative risk); SSWI: superficial wound infection; CABG: coronary artery bypass graft; CPB: cardiopulmonary bypass

	SSWI Patients (n=107)		Unadjusted RR	P-Value
	Males (n=62)	Females (n=45)		
Age (mean, SD)	55.37 (13.6)	53.78 (12.4)		<0.001
BMI (mean, SD)	27.0 (5.6)	29.3 (6.2)		<0.001
Diabetes mellitus (DM)	40 (64.5%)	26 (57.7%)	1.11	0.065
Hypertension (HTN)	40 (64.5%)	21 (46.6%)	1.38	0.045
Smoking	39 (62.9%)	2 (4.4%)	2.64	<0.001
Renal failure	14 (22.5%)	15 (33.3%)	0.67	0.21
CPB time (min/SD)	51.8 min (32)	68.4 min (39)	0.76	0.06
Re-do operation	17 (27.4%)	10 (22.2%)	1.23	0.54
Operative ICU stay (days)	15.5 days (8.2)	14.8 days (6.9)		<0.001
Surgery type				0.28
CABG	34 (54.8%)	20 (44.4%)	1.23	
Others	28 (45.1%)	25 (55.5%)		

Major risk factors

**Figure 3:**
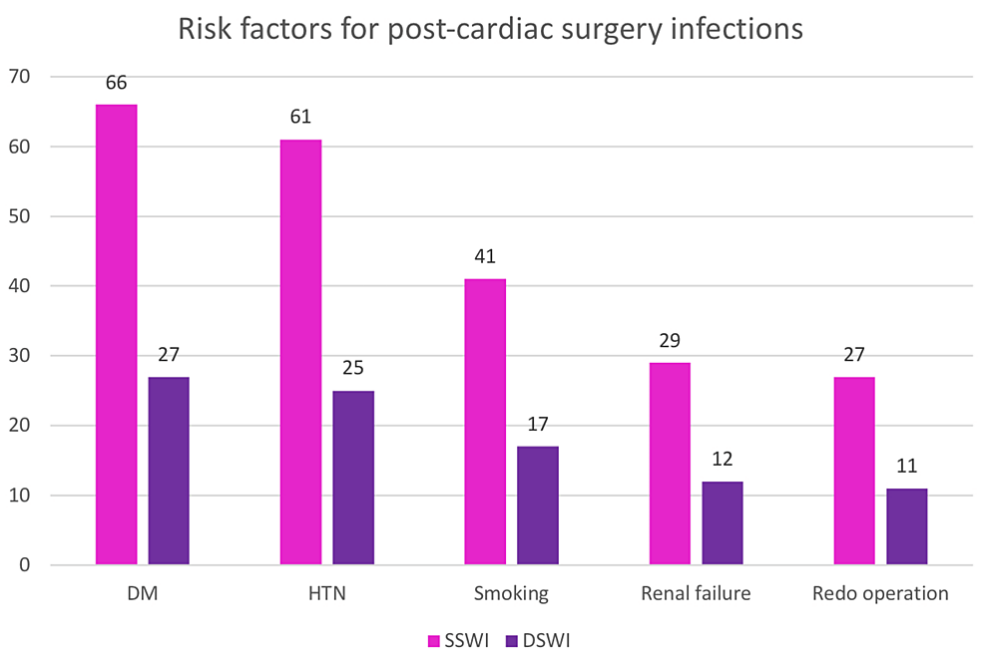
Risk factors for post-cardiac surgery infections

Major risk factors that were the main cause of post-cardiac surgery infections were diabetes, hypertension, smoking, renal failure, and re-do operation, as we can see in Table 6 and Figure 3.

**Table 6:** Risk factors for post-cardiac surgery infections DSWI: deep wound infection; SSWI: superficial wound infection

	DM	HTN	Smoking	Renal failure	Redo operation
SSWI	66	61	41	29	27
DSWI	27	25	17	12	11
Total	93 (61.5%)	86 (56.9%)	58 (38.4%)	41 (27.1%)	38 (25.1%)

## Discussion

The main aim of this study was to determine the risk factors that are involved in post-cardiac surgery infections. The study was conducted on 147 patients that had undergone cardiac surgeries of different types. Forty-four out of 147 patients were found to have developed post-cardiac surgery infections. Major risk factors involved were smoking, renal failure, DM, HTN, and prolonged ICU stay. Different studies have shown different risk factors that were involved in post-cardiac surgery infections (13.3-14%) [25,26]. Different sorts of infections like the incidence of pneumonia were also reported due to the use of damaged TEE probes with a 2.4-10.7% infection rate [27]. Different reports of different centers have reported different rates of infections like urinary tract infections (1.4%), DSWIs (1.7%), bloodstream infections (2.8%), and SSIs (5.7%) [28].

In this study, it was found that out of 389 patients that had undergone cardiac surgery, 9.4% (n= 36) patients had developed SSIs. The mortality rate was found to be 38.9% (n=14). A major factor that contributed to the development of SSIs was the pathogen attack within which Staphylococcus aureus was the main factor, infecting 12 patients (27.3%). Other factors that contributed to the development of SSIs were found to be re-incubation, incubation time greater than 24 hours, and male gender [29]. Similarly, another study had shown risk factors in the development of SSIs after cardiac surgery. The odds ratio calculated via data analysis of the study had shown that the incidence of SSIs was found to be mainly in the female population of the sample study (OR=3.5). Other factors that contributed to the development of SSIs were DM (OR=3.8) and obesity (OR=2.0). This data when compared to the control group of the same age, operation date, and type, the risk ratio was found to be 2.0 for DM, 6.2 for obesity, and 2.1 for females (sex) [30].

Similarly, another study in the pediatric ward determined the infection rate after cardiac surgery. Out of 353 patients, 55 developed 69 hospital-acquired infections (HAIs) with an incident rate of 16.4%. The most commonly occurring HAIs were surgical wounds (8%) and bloodstream infections (10%). The main pathogenic organisms were Pseudomonas sp. (16%), Enterobacter sp. (17%), and Klebsiella sp. (22%). Risk factors for HAI occurrence were found to be prolonged ICU stay, neonatal age, high complexity score, and open chest postoperatively [31].

Further online cohort studies also show that after cardiac surgery acute infection in the kidney is also a major effect. As in this study, an infection defined as a fresh infection at the surgical site or a positive blood or urine culture was the main result. In-hospital mortality, intensive care unit (ICU), stroke, and hospital duration of stay were examples of secondary outcomes (length of stay (LOS)). Acute kidney infection (AKI) in stage 1 affected 293 individuals (18.3%). Compared to 8.1% of patients without stage-1 AKI, 20.9% of patients with stage-1 AKI had an infection. Postoperative infection, ICU LOS, and hospital LOS are all independently related to stage 1 AKI. Strategies for treating AKI that emphasize prevention, early detection, and the best medical therapy may significantly reduce postoperative morbidity [32]. A supportive study shows the development of a central venous catheter-related infection (CVCRI) and the existence of a surgical site infection (SSI). There were 7557 patients included in all, and 133 SSIs (1.7%) were found. The novel finding of this study is that SSI development was 5.2 times more prevalent in individuals with CVCRI than in those without CVCRI having factors that our study has [33].

To treat life-threatening arterial bleeding, myocardial ischemia, or cardiac failure during the perioperative period, approximately 50% of patients having standard cardiac surgery need intravenous antihypertensive medication. It might be difficult to control hypertension in this situation because appropriate end-organ perfusion must be maintained while blood pressure is being reduced. Episodes of hypotension or hypertension might raise the risk of cardiac. Hypertensive urgency is defined as a significant increase in blood pressure without indications of growing target organ failure in the Seventh Report of the Joint National Committee on prevention, detection, devaluation, and treatment of high blood pressure. As evidenced by encephalopathy, myocardial infarction, pulmonary edema, angina pectoris, eclampsia, life-threatening arterial bleeding, stroke, or aortic aneurysm, a hypertensive emergency is an increase in blood pressure that is complicated by signs of impending or progressive target organ damage [34]. A further retrospective study included people who had a sternotomy during heart surgery over a five-year period. Of the 1042 patients, 25 patients (2.93%) had mediastinitis. The three primary clinical symptoms were sternal instability (30%), fever (40%), and surgical infection (80%). High C-reactive contents and hyperleukocytosis were detected biologically in 22.7% and 58.1% of patients, respectively [35]. An approach employing a database of adult patients who have had coronary artery bypass grafting surgery. The incidence rate of deep sternal wound infection was 22% throughout the entire cohort. Diabetes is one of the risk factors for deep sternal wound infection. length of surgery, use of an intra-aortic balloon pump, and obesity [36].

In the cardiac surgery population, hyperglycemia is typical and has been linked to higher death rates, postoperative length of stay, and infection rates. Although it is well known that hospitalized diabetic patients experience greater difficulties, new research in a variety of hospital settings has found that individuals with stress hyperglycemia fared worse than those with diabetes. Patients with diabetes and those with stress hyperglycemia experienced the same mortality rates. For both groups, the prevalence of severe hypoglycemia was modest (1%), and it wasn't linked to a higher death rate. Patients with diabetes and those who had stress hyperglycemia experienced a similar incidence of other complications [37].

Except for the above-mentioned studies, we should also consider these factors along with microbial control, as studies have shown that one of the most important post-surgical healthcare issues is septic purulent nosocomial infections (SPNI). These are linked to higher mortality, morbidity, and admission fees. Finding out how widespread the illnesses are in hospitals is important. A retrospective analysis was performed on a cross-sectional database that had 1189 records of heart surgery patients at a multi-profile hospital. Following heart surgery, there were 317.57 SPNI incidents as opposed to the 15.02 officially recorded. In the study of 418 instances with SPNI, the most frequent infections were respiratory tract infections (19.14%), surgical site infections (32.06%), and infections associated with other illnesses (23.18%). Twenty-eight types of bacteria, including gram-positive (61.92%) and gram-negative (38.08%) ones, make up the etiological structure. More urgent steps must be taken to prevent and control these infections given the relatively high occurrence of SPNI and its effects [38].

Study limitations

We are aware of a few study limitations that should be addressed. First, these data are based on a unicentric hospital; hence, the results cannot be generalized. Then, the study observations were for not more than 30 days after discharge.

## Conclusions

In conclusion, this study has demonstrated major risk factors that are involved in the occurrence of post-cardiac surgery infections like smoking, diabetes mellitus, prolonged ICU stay, sex, more than one operation during a single stay, etc. Heart surgery and nosocomial infections (cardiac) are serious public health concerns that harm patients. The entire picture that is shown demonstrates that there is a stronger impact on postoperative morbidity, mortality, and longer hospital stays. Global expenditures may be reduced by corrective and preventive actions that lessen the impact of nosocomial extracardiac infections. In the future, the contribution of various other factors involved in the occurrence of surgical site infection, best practices, and methods should be studied and implemented to prevent the occurrence of post-cardiac surgery infections. Various clinical evaluations can still be utilized to prevent these sorts of infections, which will decrease the mortality rate.
